# Clinical Profile of Pediatric Tuberculosis in a Tertiary Hospital in Northeast India: A Retrospective Analysis

**DOI:** 10.7759/cureus.38660

**Published:** 2023-05-07

**Authors:** Rosina Ksoo, Himesh Barman, Manisha De, Donboklang Lynser, Sourabh G Duwarah, Clarissa Lyngdoh

**Affiliations:** 1 Paediatrics, North Eastern Indira Gandhi Regional Institute of Health and Medical Sciences, Shillong, IND; 2 Radiology, North Eastern Indira Gandhi Regional Institute of Health and Medical Sciences, Shillong, IND; 3 Paediatrics and Neonatology, Akanksha and Ayursundra Hospital, Guwahati, IND; 4 Microbiology, North Eastern Indira Gandhi Regional Institute of Health and Medical Sciences, Shillong, IND

**Keywords:** hydrocephalus, tuberculous meningitis (tbm), extrapulmonary tuberculosis (eptb), disseminated tuberculosis, pulmonary tuberculosis, acid fast bacilli, pediatrics, tuberculosis

## Abstract

Context

Tuberculosis (TB) is India’s major public health problem. The profile of childhood TB in the northeast region of India is still limited.

Aim

To analyze the clinical, radiological, and bacteriological profiles of children with TB at a tertiary health care facility.

Materials and methods

A three years retrospective descriptive analysis of children admitted to a tertiary centre with TB before the introduction of cartridge-based nucleic acid amplification test (CBNAAT) for testing. Children below 18 years who were admitted from 2012 to 2014 and were diagnosed with TB were included. Relevant data were extracted in a predesigned format and entered into a Microsoft Excel sheet. Descriptive statistic was used for analysis. The results of variables are given in proportions and means and a Chi-square test was done for the test of significance using Epi-info tools. The study was done after getting ethical approval from the institute.

Results

A total of 150 children were included in the analysis with a Male: Female ratio of 1.1:1. A majority of the cases were under five years (n=46) and 11 to 15 years old (n=45) with a mean age of 9.3 ± 4.4 years. Fever was a common presentation (70%). Disseminated TB was seen in 31.3%, isolated central nervous system (CNS) TB was found in 30.6%, and all CNS TB with dissemination was found in 46 cases (40.7%) making extra-pulmonary TB a common finding in our study (83.3%). Isolated pulmonary TB was seen in 16.7% and total pulmonary cases along with dissemination was seen in 60 cases (40%). A bacteriological diagnosis was made in 23%. Overall mortality was 9.3%, out of which mortality in CNS TB was 13% with a p-value of 0.004 as compared to mortality other than CNS TB which was significant and mortality in under-five years was significant with a p-value of 0.001.

Conclusions

Pulmonary and extra-pulmonary were both causes of admission in the pediatric age group. We found that extra-pulmonary TB was the most common cause of admission in children, with CNS manifestation and disseminated TB, being the most common presentations and significant mortality was seen in under-five years and in children diagnosed with CNS TB.

## Introduction

Tuberculosis (TB) is a communicable disease caused by *Mycobacterium tuberculosis* and is a major health problem. It is one of the top 10 causes of death worldwide and the leading cause of death due to a single infectious agent, contributing to 1.4 million deaths in 2019. The South East Asia (SEA) region accounts for 44% of the global burden of TB. India is among the eight high-burden countries with TB which contributes to 2/3 of the world’s TB cases. An estimated 10 million people were diagnosed with TB and children less than 15 years account for 12% of this number in 2019 [1]. Northeast states in India are among the high-burden states with TB in the country [2].

TB is broadly classified as pulmonary and extra-pulmonary TB. Children and adolescents with TB pose difficulty in diagnosis and hence their subsequent treatment. The paucibacillary nature of the disease in children makes microbiological diagnosis difficult. This is particularly true for extra-pulmonary TB where microbiological yields are even lower. This also results in gross under-reporting and underestimation of the burden of pediatric TB.

There is a dearth of published data about the presentation and organ involvement pattern of children with TB from the northeast region of India. We aim to bridge this gap by analyzing the clinical, radiological, and bacteriological profiles of children with TB at a tertiary health care facility. Documenting the presentation and spectrum of childhood TB is likely to aid the suspicion and early diagnosis of TB in children. Our area is a resource-constraint area and this data pertains to the period before the cartridge-based nucleic acid amplification test (CBNAAT) was adopted as the microbiological test for the diagnosis of pediatric TB.

## Materials and methods

We are a tertiary care referral centre catering to patients from all over the northeastern states of India. Our study was a retrospective descriptive study. We included all children below 18 years by consecutive sampling method, who was admitted with the diagnosis of TB from January 2012 to December 2014 in the Department of Pediatrics. This study was done during the period before the introduction of CBNAAT for microbiological diagnosis of pediatric TB. The study was conducted after getting ethical approval from the Institute's Ethics Committee (NEIGR/IEC/M15/F31/2021).

Relevant data were extracted and entered in a predesigned format in a Microsoft Excel sheet that included signs and symptoms, household contact with TB, and Bacille Calmette-Guerin (BCG) vaccination status. Radiological studies, Mantoux tests, and bacteriological studies were reviewed for all children diagnosed with TB. Children diagnosed with TB from the outpatient department (OPD) were excluded from the study because of inadequate data.

Children were diagnosed with probable TB based on national guidelines [3]. Presence of any or all of these: (a) Persistent fever and/or cough > two weeks; (b) loss of weight/no weight gain; (c) history of contact with infectious TB cases, including positive tuberculin skin test (TST) and chest X-ray suggestive of TB. The severity of tuberculous meningitis can be classified based on a system devised by the British Medical Research Council: Stage I patients are fully conscious, rational, and do not have neurologic signs; Stage II patients are confused or have neurologic signs such as cranial nerve palsy or hemiparesis and Stage III patients are comatose or stuporous with more severe neurologic signs [4].

Descriptive statistics were used for analysis. The results of variables are given in proportions and means and a Chi-square test was done for the test of significance using Epi Info tools (United States Centers for Disease Control and Prevention (CDC), Atlanta, Georgia).

## Results

A total of 163 children were diagnosed with TB during the study period. Out of these 150 children were included in the final study and 13 were excluded due to insufficient data or did not meet the criteria guidelines for diagnosis.

**Figure 1:**
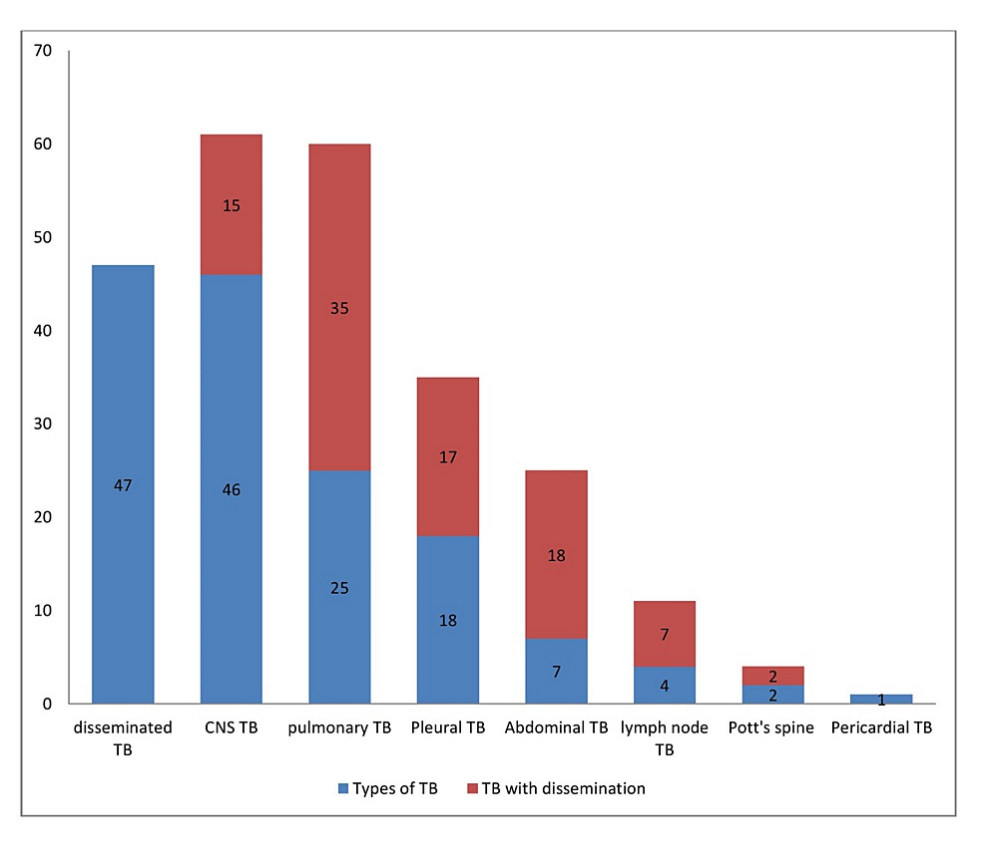
Distribution and types of pediatric tuberculosis TB: tuberculosis

**Figure 2:**
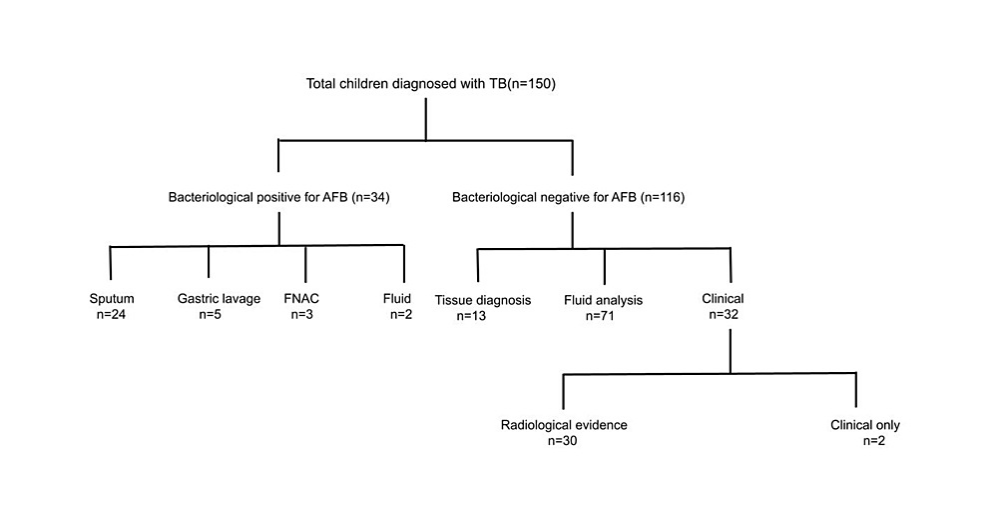
Distribution of cases based on the mode of diagnosis TB: tuberculosis; AFB: acid-fast bacilli; FNAC: fine needle aspiration cytology

Of the 150 cases, 53.33% were boys. The mean age at presentation was 9.3 ± 4.4 years. The most common age group who got admitted were under five years (n=46) and 11-15 years (n=45), followed by 6-10 years (n=37) and 16-17 years (n=22). The clinical features at the time of presentation are shown in Table 1. The most common type of TB in our study was disseminated TB (31.3%) and isolated pulmonary TB was found in 16.7% (n=25). The rest of the distribution of the type of TB cases in our study is shown in Figure 1. A positive history of contact in the family was present in 26.7%. TST could be traced in 45 children from the 150 cases, out of which 18 (40%) had a reactive skin test. Malnutrition was seen in 45.3% of cases. We have a total bacteriological yield from both pulmonary and extra-pulmonary TB of 34 cases (22.67%), and diagnosis based on radiological findings including chest X-ray, computed tomography (CT) and magnetic resonance imaging (MRI) was helpful in 20% of cases (Figure 2). Adenosine deaminase (ADA) was sent for all children with pleural, peritoneal, or CNS TB if the parents could afford it and were above the diagnostic levels (>60 IU/L) in 41 (93.2%) out of 44 children. Erythrocyte sedimentation rate (ESR) was high (>40 mm in the first hour) in 78.7% of children. The data on BCG vaccination status or BCG scar was not available. CBNAAT was not done as it was unavailable during the study period.

**Table 1:** Clinical features at presentation, n=150 (%) ATT: anti-tubercular treatment

1	Fever	106 (70.7%)
2	Cough	65 (43.3%)
4	Already on ATT	35 (23.3%)
5	Weight loss and decreased appetite	32 (21.3%)
6	Chest pain and dyspnea	29 (19.3%)
7	Vomiting	25 (16.7%)
8	Altered sensorium	24 (16%)
9	Seizure	24 (16%)
10	Abdominal pain	24 (16%)
11	Meningeal irritation	20 (13.3%)
12	Headache	19 (12.7%)
13	Lymphadenopathy	13 (8.7%)
14	Abdominal distension	9 (6%)
15	Anasarca	8 (5.3%)
16	Focal neurological deficit	8 (5.3%)
17	Cranial nerve Palsy	7 (4.7%)
18	Blurred vision	6 (4%)
19	Hemoptysis	5 (3.3%)
20	Loose stool, severe acute malnutrition	4 each (2.7% each)
21	Psoas abscess	3 (2%)

Disseminated TB was the most common presentation of TB seen in our study (Figure 1). The mean age was 10.5 ± 7.9 years, with nine (19%) children under five years, 13 (27.7%) between the age group of 6-10 years and 25 (53%) children above 10 years. The youngest child was a four-month-old with miliary tuberculosis. The smear positivity was seen in 15 out of 34 cases (44%). Weight for age ≤3rd percentile was found in 23 out of 33 children (69.7%) with a mortality of three (21.4%).

TB with CNS disease was also another common involvement in our study. The mean age at presentation for isolated CNS TB was 6.2 years and for all CNS TB with dissemination was 10.2 ± 8 years. The clinical and radiological features of isolated CNS TB and all CNS TB with dissemination are shown in Table 2.

**Table 2:** Clinical and radiological findings in CNS TB CNS: central nervous system; CSF: cerebrospinal fluid; TB: tuberculosis; ADA: adenosine deaminase; VP: ventriculoperitoneal

Parameters	Isolated CNS TB (n=46, 30.67%)	All CNS TB cases (n=61, 40.67%)
Mean age (years)	6.2	10.2 years
Median age (years)	6	7
< 5 years	22 (46.7%)	24 (39.3%)
6-10 years	11 (22.4%)	16 (26.2%)
11-17 years	13 (28.9%)	21 (34.4%)
M:F	1.4:1	1.2:1
Fever	31 (68.9%)	43 (70.5%)
Altered sensorium	19 (42.2%)	23 (37.7%)
Seizure	19 (41.3%)	23 (37.7%)
Vomiting	17 (36.9%)	23 (37.3%)
Headache	12 (26.7%)	18 (29.5%)
Positive contact with TB	11 (24.4%)	15 (24.6%)
Focal neurological deficit	7 (15.5%)	7 (11.5%)
blurred vision	4 (8.9%)	5 (8.2%)
Dizziness	1 (2%)	1 (1.6%)
CSF lymphocytic predominance	18 (37.8%)	28 (45.9%)
CSF ADA >10	10 (20.8%)	14 (23%)
Stage I		9 (14.8%)
Stage II		21 (34.4%)
Stage III		31 (50.8%)
Neuroimaging		
Hydrocephalous	28 (62.2%)	35 (57.4%)
Meningeal enhancement	23 (51%)	29 (47.5%)
Tuberculoma	18 (40%)	22 (36%)
Infarcts/vasculitis	13 (28.2%)	15 (24.6%)
VP shunt insertion	22 (48.9%)	25 (41%)
Mortality	6 (13.3%)	8 (13.1%)

Clinical features of isolated pulmonary TB along with all pulmonary involvement are shown in Table 3. Endobronchial TB was seen in four children, two had caseating granuloma on bronchoscopy and the other two showed tracheal narrowing on radiological imaging.

**Table 3:** Clinical findings in pulmonary TB and pleural TB TB: tuberculosis; AFB: acid-fast bacilli

Clinical features	Pulmonary TB, n= 25 (16.7%)	All pulmonary TB n=60 (40%)	Pleural TB, n= 18 (12%)	All pleural TB n=35 (23%)
Mean age (year)	9.8	10.2	12.2	12.9
Median age	10	10	11.5	13
0-5 years	7 (28%)	13 (21.3%)	5 (27.8%)	7 (20%)
6-10 years	5 (20%)	17 (27.9%)	2 (11.1%)	6 (17.1%)
11-17 years	13 (52%)	31 (50.8%)	11 (61.1%)	22 (62.9%)
M:F ratio	7:18	15:21	13:5	18:17
Fever	20 (80%)	47 (77%)	13 (72.2%)	27 (77.1%)
Cough	19 (76%)	39 (63.9%)	15 (83.3%)	25 (71.4%)
Pain abdomen	3 (12%)	12 (19.7%)	4 (22%)	9 (25.7%)
Weight loss	7 (28%)	15 (24.6%)	2 (11%)	8 (22.9%)
Breathing difficulty	7 (28%)	14 (23%)	12 (66.7%)	17 (48.6%)
Hemoptysis	4 (16%)	5 (8.2%)	0	0
H/o contact with TB	7 (28%)	20 (32.7%)	4 (22%)	9 (25.7%)
Chest X-ray suggestive of TB	16 (64%)	38 (62.3%)	16 (88%)	22 (62.9%)
Sputum/gastric lavage positive for AFB	14 (56%)	29 (47.5%)	0	3 (8.6%)
Tissue/fluid positive for AFB	2 (8%)	2 (3.2%)	0	1 (2.9%)
Mortality	2 (8%)	4 (6.6%)	1 (5.5%)	3 (8.6%)

Isolated abdominal TB was seen in only eight cases (5.3%) but a total of 26 children (17.3%) had associated abdominal involvement. The mean and median ages were 11 years. Positive contact with TB was present in 31%. The most common presenting complaints were pain abdomen at 54%; fever, cough and significant weight loss at 31% each; abdominal distension at 27%; and loose stools at 11.5%. The commonest ultrasound findings were ascites in 61.5%; abdominal lymphadenopathy in 31%; peritoneal thickening in 11.5%; ileocaecal thickening in 7.7%; septations, granulomatous peritonitis and omental caking were seen in 4% each. In ascitic fluid analysis, there was lymphoid predominance in 61.5%. ADA levels were high at 31%. Acid-fast bacilli (AFB) was present in ascitic fluid and from fine needle aspiration cytology (FNAC) of abdominal lymph nodes in 7.7%.

Tubercular lymphadenopathy was seen in 11 cases (7.3%). The mean and median age of presentation is 9.7 and nine years respectively. FNAC done in eight cases were suggestive of caseating granulomatous lymphadenitis and three (37.5%) were positive for AFB.

Bone involvement in the form of Pott’s spine was seen in four children (2.7%) and of which two were disseminated TB and both were 1.5 years old. The mean age was 3.7 years. There was positive contact in two cases. MRI spine was done in two and ultrasound in one which was suggestive of TB and psoas abscess. The fourth one was already diagnosed with TB and was positive for AFB in gastric lavage on presentation. All had presented with back swelling. One had a weakness in the lower limb.

A rare form of TB involving the pericardium was also seen in an 18-month-old child.

Mortality was seen in 9.3%. Overall mortality due to CNS TB was 13% which was significant. Mortality in those under five years was significant as compared to those older than five years (Table 4).

**Table 4:** Comparison of mortality TBM: tuberculous meningitis

		Mortality (n-14)	ᵡ^2^	P value
1	Under 5 years	7	2.71	0.001
	>5 years	7		
2	Male	5	1.92	0.16
	Female	9		
3	TBM	10	7.888 (Yates correction)	0.004
	Non-TBM	4		
4	TBM stage 3	6	0.02 (Yates correction)	0.88
	TBM stage 1-2	4		
5	Old cases	5	1.014	0.31
	New cases	9		
6	Pulmonary	4	0.39 (Yates correction)	0.52
	Extra pulmonary	10		

## Discussion

We analyzed the clinical-radiological and bacteriological profile of 150 patients and found that the majority of our affected children were under-five years (30.7%) which was similar to other studies [5-11] and age group between 11 and 15 years (30%) which when compared to a study done by Mazta et al. [12] also found that this was the most common age group in their study and their study had limited number of children under-five years. This finding reflects the ongoing infection with TB in the household and robust contact tracing and initiation of isoniazid prophylaxis for children under-five years should be strengthened. Children over 10 years were seen in 44.7% which was similar to findings by Arora et al. There were still 24.6% of children who were affected in the age group of 6-10 years in our study which was comparable to other studies by Arora et al. and Dhaked et al. [13,14]. Our findings were consistent with the study done by Marais et al. [15], where they identified the two vulnerable age groups for the development of TB: in younger children under five years and in adolescents. The mean age at presentation in our study was 9.3 years and the median age of 9.7 years, which was comparable to other studies [12,14,16,17]. There were more males than females, with a male: female ratio of 1.1:1 which was comparable to others [6,8,10,18,19].

Isolated pulmonary TB cases were seen in 16.7% and with dissemination was seen in 40% of cases, which makes extra-pulmonary TB with dissemination was common presentation found in 83.3% of our study, this high number could be attributed to the referral bias and also our centre being a tertiary centre that has facilities for an intensive care set-up for children. This could also be explained by the fact that we only included children who were sick and required admission to the study. Extra-pulmonary TB was the most common finding in other studies as well [14,19-22] even when their study populations included children less the 15 years. This also highlights the seriousness and need for admission in children suffering from extra-pulmonary TB. Conversely, this also gives us a clue to work up any children who require admission for TB for dissemination and extra-pulmonary TB.

The history of contact was found in 27.7% which was similar to other studies [7,16,18,21,23]. Newly diagnosed cases in our study were 76.7% which was less than in other studies [20,24] probably because our study population included only children who were sick and referred for further management. TST could be traced in only 45 children from the total 150, out of which 18 (40%) had reactive skin test which was comparable with some studies [21,25] but less than others [5,7,18,23] likely because of the retrospective nature of our study and insufficient data. Malnutrition was seen in 45% of children which was similar to the finding by Goyal et al. [25] even though their study population is less than 15 years, but this finding was less as compared to 93% by Thanvi et al. [21], probably because our study population included older children as well upto the age of 18 years. BCG status could not be extracted from our study due to inadequate data. Raised ESR in our study was 78.7%, similar to the study done by Goyal et al. [25]. Mortality was seen in 14 cases (9.3%), similar to other findings [9,21,22] and 13.1% of death due to CNS TB is comparable to a 10-year retrospective comparative analysis done by Miftode et al. [26] again re-emphasizing the importance of early diagnosis, actively ruling out disseminated TB and proper treatment of all TB cases.

Disease types

TB with CNS involvement was seen in 40.6% of cases similar to Gosai et al. [6]. The majority were below five years (39.3%) with a median age of seven years, this was comparable with other studies [8,9,10,11,27]. Hydrocephalus was the most common neuroimaging finding which was similar to other studies [8-10], followed by meningeal enhancement which was also comparable to other studies [8-11]. Ventriculoperitoneal (VP) shunt was done in 41% of cases compared to 81% by Yaramis et al. [8], probably because more than 50% of our cases presented in stage 3 and were referred for complications and further management.

Amongst pulmonary TB, the median age was 10.2 years vs 5.4 years in a study by Goyal et al. [25]. We could demonstrate a similar bacteriological confirmation as compared to other studies [17,23,24,28]. We got more positive results for AFB from sputum (82%) as compared to gastric lavage, which is different as compared to the study by Jiménez et al. [28] which was 41.2% from induced sputum and 47.1% from gastric lavage. This difference could be because of their sample size (n=22) and they included cases with only pulmonary TB and our yield could have been improved with the use of CBNAAT. Pleural TB was found in 12% of isolated cases which is comparable with others [6,13,14,16,20], and 23% of total cases with pleural involvement which is also similar to that by Mazta et al. [12].

Isolated cases of abdominal TB were seen in 5.3% of our cases which is similar to other findings [12,16,19] but when we take all the cases with abdominal involvement, it is more (17.3%) than other studies [6,14,18,21]. The mean age of abdominal cases in our study is younger (11 years) than that found by Lin et al. [29] which is 14 years. The contact history is similar to that of Shah et al. [18]. Pain abdomen (54%) was similar to other studies [6,18]. In ultrasound abdomen peritoneal involvement (11.5%) was similar to that by Shah et al. [18] but less than the study by Lin et al. (66%) [29] probably because of the small sample size (n=10). Mycobacterium TB from ascitic fluid was isolated in 7.7% which is less than that found by Lin et al. (20%) [29] probably again due to their small sample size. Hence abdominal TB, although difficult to diagnose, should be suspected in any patient with abdominal pain, early imaging plays an important role in the diagnosis since the bacteriological yield is poor and they often present with disseminated or extra-abdominal TB.

Tuberculous lymphadenopathy was seen as similar to other findings [6,30] but less than others [12,13,16,19] probably because we did not include the stable patients treated from our outpatient department. Pott's spine was seen in 2.7% of our cases which is comparable to other studies [12,19,20,30]. Pericardial TB is a rare finding in our study (0.67%) which is comparable to another study [30].

The strength of our study is that it's the first study that profiles pediatric TB in the northeast region, highlighting the high incidence of CNS TB in children and reiterating the poor outcome despite adequate care. All microbiological and pathological diagnoses were attempted in all cases and the diagnosis was made as per the national guidelines.

Our study was limited by its retrospective nature. We included only in-patients for our study and being a tertiary care centre, there was a referral bias which hampered the description in our cohorts. CBNAAT was not available for diagnosis during the study period, which could have further improved our microbiological yield.

## Conclusions

We documented that both pediatric pulmonary and extra-pulmonary TB were common in our region. The high burden of CNS TB and disseminated TB results in large proportions presenting with extra-pulmonary TB in our study. A malnutrition rate of 45% was seen which may have contributed to the high dissemination rate. The most common age group that required admissions and suffered mortality was under-five years, underscoring the fact that younger children are the most vulnerable group. Prevention of serious disease in this age group can be done by robust contact tracing for household members and initiating isoniazid prophylaxis for under-five years wherever required. Our total bacteriological yield was 23% justifying attempts to make a microbiological diagnosis in all pediatric TB suspects. Used of CBNAAT would have likely increased the yield.
